# The relationship of postocclusive reactive hyperemia assessed by the plethysmographic perfusion index to lactate clearance: a new piece in the unsolved puzzle of tissue perfusion and oxygenation in septic shock

**DOI:** 10.5935/2965-2774.2023.Edit-2.v35n2-en

**Published:** 2023

**Authors:** Arnaldo Dubin

**Affiliations:** 1 Servicio de Terapia Intensiva, Sanatorio Otamendi - Buenos Aires, Argentina

Septic shock is commonly characterized by the lack of coherence between systemic hemodynamics and microcirculation.^(1)^ The optimization of systemic cardiovascular variables frequently fails to improve the outcome of septic patients. Since the final goal of resuscitation should be the normalization of tissue perfusion and oxygenation, there is a growing interest in the monitoring of microvascular flow. Unfortunately, few tools for this goal are available in the clinical arena.

Alterations in cutaneous perfusion are typical manifestations of every type of shock. Although sophisticated methods might be used for the study of skin microcirculatory disorders, clinical evaluation is still a key approach.^(2)^ The presence of mottling and its severity are strongly associated with mortality in patients with shock.^(3)^ The capillary refill time is also a useful, inexpensive, and universally accessible method. It provides relevant prognostic information and can successfully guide the resuscitation of patients with septic shock.^(4)^ The problem is that measurement of capillary refill time is poorly reproducible. Even after careful standardization and training, the interand intraobserver variability of the method is wide.^(5)^ The capillary refill time changes according to the environmental temperature, age, sex, and skin characteristics.^(6)^ Another valuable tool for the evaluation of cutaneous perfusion is the perfusion index (PI), which is derived from the analysis of the plethysmograph waveform of the pulse oximeter.^(7)^ The PI is the ratio between the pulsatile component (arterial compartment) and the nonpulsatile component (venous and capillary blood) of the light reaching the detector of the pulse oximeter. Thus, the reduction in the pulsatile component by peripheral vasoconstriction decreases the ratio and thus the PI. In healthy volunteers, the values of PI have a highly skewed distribution, and they range from 0.3 to 10.0. Nevertheless, PI correlates with the core-to-toe temperature difference. In critically ill patients, a PI value below1.4 reflects the presence of poor peripheral perfusion.^(7)^ Perfusion index can be used for the assessment of fluid responsiveness during a maneuver of passive leg raising.^(8)^ Moreover, the dynamic response of the PI to a vascular occlusion test (VOT) allows the study of reactive hyperemia, which is the ability to recruit the microcirculation after an ischemic challenge.

In this issue of Critical Care Science, Miranda et al. publish a new contribution to our understanding of this issue.^(9)^ Previously, Menezes et al. showed that patients with septic shock, compared to nonseptic controls, took longer to reach the peak PI after a VOT (70 [53 - 92] *versus* 48 [36 - 60] sec).^(10)^ Although the maximal variation in the PI (∆PI) was similar in the two groups (71 [32 - 125] *versus* 79 [30 - 137] %), the change in the first 60 sec after the VOT (∆PI_0-60_) was lower in the septic group (1 [-19 - 40] *versus* 39 [6 - 75] %). In contrast, the ∆PI in the following 60 sec (∆PI_60-120_) was similar (48 [18 - 98] *versus* 43 [18 - 93] %). ∆PI_0-60_ and ∆PI_60-120_ are probably linked to mechanosensitive and metabolic responses.^(11)^ In a further study in patients with septic shock, the authors found that nonsurvivors took longer to reach the maximal PI.^(12)^ Paradoxically, nonsurvivors had a higher ∆PI than survivors, which was completely explained by differences in ∆PI60-120. Consequently, a peak ∆PI > 62% was a strong predictor of mortality. In summary, the mechanosensitive response is decreased in septic patients compared to nonseptic controls, but the metabolic response is higher in nonsurvivors than in survivors with septic shock. Interestingly, both studies showed a positive correlation between peak ∆PI and vasopressor dose, whose underlying mechanism involves alterations in adrenergic regulation.^(10,12)^

Now, Miranda et al.^(9)^ studied a series of patients with septic shock who maintained hyperlactatemia after resuscitation within the first day of diagnosis. Their goals were to confirm the prognostic value of the test and to assess its relationship with lactate level. The strengths of the study were the prospective and multicenter design, as well as the inclusion of a relatively large number of patients. Unfortunately, there were many missing data. Patients with a ∆PI peak > 62% had a higher mortality (66.1 *versus* 38.5%), more alterations in peripheral perfusion, and nonsignificant trends toward higher lactate levels, lower lactate clearance, and norepinephrine doses than patients with a ∆PI peak < 62%. A high ∆PI peak identified a group of patients with a more severe condition. Although the value of a high ∆PI peak as a predictor of mortality was confirmed, the study failed to show any clear associations with lactate clearance. This result is not unexpected considering the multiple sources of hyperlactatemia in septic patients.

The findings of Miranda et al.^(9)^ and Menezes et al.^(10,12)^ are not necessarily paradoxical and could reflect the intricate abnormalities of reactive hyperemia and tissue oxygenation in septic shock. The delayed response to reach the peak PI, in comparison to nonseptic patients, could be an expression of altered reactive hyperemia. On the other hand, a higher ∆PI in nonsurviving patients with septic shock might show the payment of an increased oxygen debt acquired during the VOT.

In summary, PI is a useful tool for the monitoring of tissue oxygenation in critically ill patients. Its combination with a VOT not only provides new insights into the complexity of microvascular recruitment but also improves the prognostic ability. Even though the study from Miranda et al.^(9)^ adds new information to the previous findings of Menezes et al.,^(10,12)^ the interpretation and the clinical usefulness of the test are not straightforward. Further research is needed to completely understand the meaning and mechanisms of these interesting findings.
